# Role of atrial natriuretic peptide in mediating the blood pressure-independent natriuresis elicited by systemic inhibition of nitric oxide

**DOI:** 10.1007/s00424-014-1557-4

**Published:** 2014-06-24

**Authors:** Leszek Dobrowolski, Marta Kuczeriszka, Alexander Castillo, Dewan S. Majid, L. Gabriel Navar

**Affiliations:** 1 Department of Physiology, Hypertension and Renal Center of Excellence, Tulane University Health Sciences Center, New Orleans, LA USA; 2 Department of Renal and Body Fluid Physiology, M. Mossakowski Medical Research Centre, Polish Academy of Sciences, A. Pawińskiego 5 St., 02-106 Warsaw, Poland

**Keywords:** Arterial pressure, Renal haemodynamics, Sodium excretion, Medullary blood flow, Cortical blood flow, Glomerular filtration rate, Anantin

## Abstract

While it is clearly recognized that increased intrarenal nitric oxide (NO) levels elicit natriuresis, confounding data showing that systemic nitric oxide synthase inhibition (NOSi) also increases sodium excretion (U_Na_V) poses a conundrum. This response has been attributed to the associated increases in arterial pressure (AP); however, the increases in AP and in U_Na_V are temporally dissociated. The changes in regional renal haemodynamics induced by NOSi could also contribute to the alterations of U_Na_V. To evaluate the roles of AP and non-AP mechanisms mediating the natriuresis, *N*_ω_-nitro-l-arginine methyl ester hydrochloride (L-NAME) was infused i.v. at doses ranging from 5 to 50 μg/kg/min in anaesthetized rats. U_Na_V, perfusion of the cortex (cortical blood flow, CBF) and medulla (medullary blood flow, MBF) with laser-Doppler flowmetry and glomerular filtration rate (GFR) were measured. U_Na_V increased from 0.6 ± 0.2 to 1.6 ± 0.1 μmol/kg/min (*P* < 0.05) with the lower nonpressor doses. With the higher doses, AP increased from 116 ± 4 to 122 ± 4 mmHg and U_Na_V increased from 1.1 ± 0.3 to 3.3 ± 0.7 μmol/min/g (*P* < 0.002). U_Na_V increased similarly in a group where renal AP was maintained at baseline levels. The associated reductions in CBF (17 ± 5 and 38 ± 5 %) and MBF (27 ± 6 and 52 ± 6 %) would be expected to attenuate rather than contribute to the natriuresis. Plasma atrial natriuretic peptide (ANP) concentrations increased significantly following NOSi. Anantin, a natriuretic peptide receptor-A blocker, prevented or reversed the L-NAME-induced natriuresis without altering the L-NAME-induced changes in AP or CBF. The results indicate that increased ANP and related natriuretic peptides mediate the AP-independent natriuresis, at least partly, elicited by systemic L-NAME infusion and help resolve the conundrum of natriuresis during systemic NOSi.

## Introduction

Endogenously released nitric oxide (NO) plays an important role in the control of vascular and tubular function in the kidney [19, 27]. The contribution of endogenous NO to renal function has been established, in part, from the responses to l-arginine analogues that inhibit nitric oxide synthase (NOS) and elicit renal vasoconstriction in dogs and rodents [3, 9, 21, 23, 29, 31]. Furthermore, systemic infusion of NOS inhibitors is followed not only by a decrease in renal blood flow (RBF) but also by a prominent increase of mean arterial pressure (AP), whereas the intra-arterial administration leads mostly to renal vasoconstriction.

In contrast to the renal vasoconstriction observed consistently, the effects of NOS inhibitors on sodium excretion are variable and appear to depend on the route of infusion. Systemic infusion has been shown to induce natriuresis and diuresis [14, 15, 29]; these responses are opposite to the effects observed following intrarenal administration of NOS inhibitors where antinatriuresis has been observed [7, 20, 21]. Moreover, renal arterial infusion of an NO donor increases sodium excretion, demonstrating that the direct effects of NO are natriuretic and diuretic [20, 21]. These effects are not consistently associated with changes in glomerular filtration rate (GFR), indicating direct effects on tubular transport function [19]. Studies on epithelial transport have also demonstrated that NO exerts direct inhibitory effects on sodium transport in renal tubules [27]. A satisfactory explanation for the contrasting effects of systemic and direct NOS inhibition on sodium excretion and urine flow is not apparent, but the conundrum suggests that an indirect mechanism activated by the associated cardiovascular responses is responsible for the natriuresis that occurs during systemic NOS inhibition.

The increases in sodium excretion following acute systemic NOS inhibitor administration have often been attributed to the associated increases in AP [10, 13, 14, 19]. When a NOS inhibitor is given systematically, it elicits increases in AP with simultaneous decreases in renal plasma flow and cortical blood flow (CBF) but there is a distinct delay in diuresis and natriuresis [3, 4]. Thus, the natriuresis appears to be temporally dissociated from the blood pressure response and probably depends on multiple factors. Shahid et al. [36] reported that the natriuretic effect of NOS inhibition in mice is mediated, at least in part, by the concomitant generation of TNF-α. The increases in urine flow and sodium excretion could also depend on additional mechanisms and factors such as increased release of atrial or brain natriuretic peptide (ANP, BNP) or CYP-450-dependent arachidonic acid derivatives. Importantly, Leskinen et al. [17] reported that *N*_ω_-nitro-l-arginine methyl ester hydrochloride (L-NAME) administration increased plasma levels of immunoreactive ANP, but not BNP, thus providing a mechanistic link between the cardiovascular responses to NOS inhibition and the natriuresis elicited by an augmented ANP secretion.

In the present study, we evaluated the effects of a nonselective NOS inhibitor (L-NAME) on renal sodium and potassium excretion using variable doses including subpressor and slightly pressor without and with control of renal perfusion pressure. Independent of AP effects, L-NAME clearly decreased RBF. Because the changes in renal cortical and medullary blood flow (MBF) induced by NOS inhibition could contribute to the altered water and ion excretion [22], we used laser-Doppler flowmetry to follow separately the changes in regional blood perfusion in the renal cortex and medulla. In further experiments, we evaluated the role of ANP or BNP in mediating the increases in urine flow and sodium excretion by administering the natriuretic peptide receptor-A (NPR-A) competitive receptor blocker, anantin [6, 8, 26, 41], during administration of L-NAME. Finally, we measured the changes in plasma ANP concentration in response to NOS inhibition. We postulated that the L-NAME-mediated natriuresis is dissociated from the AP responses, still occurs in the absence of increases in AP and is prevented or attenuated by NPR-A blockade.

## Materials and methods

This protocol was approved by the Institutional Animal Care and Use Committee of Tulane University Health Sciences Center. Male Sprague Dawley rats (Bwt 280–340 g), fed a normal rat diet (TD 90229, Harlan-Teklad) with free access to water until the day of experiment, were anaesthetized with Inactin (thiobutabarbital sodium, Sigma, Saint Louis, USA) at 100 mg/kg i.p. and placed on a heated surgery table to maintain rectal temperature at 37 °C. A polyethylene tube was placed in the trachea, and the rats were prepared for renal clearance experiments as previously described [4, 25]. The femoral vein was cannulated for fluid infusions, and the femoral artery was catheterized for systemic arterial blood pressure (AP) and heart rate measurements.

The left kidney was exposed from a subcostal flank incision and placed in a plastic cup similar to that used for micropuncture [25]. For timed urine collections, a catheter was introduced into the ureter and passed to the pelvis. The renal artery was separated from the renal vein to enable placement of a noncannulating flow probe, 1 mm in diameter, connected with a Transonic flowmeter (type T106, Transonic System Inc., Ithaca, NY, USA) for measurement of the total RBF. RBF was measured only in experiments where placement of the probe did not cause technical problems, but the surgical procedures were performed in every rat. The blood perfusion rates of the renal cortex and outer medulla were recorded separately using the laser-Doppler Periflux 4001 system (Perimed AB, Jarfalla, Sweden) [4]. The CBF was measured using a PF 408 probe placed on the kidney surface. The outer MBF was measured as laser-Doppler flux using a needle probe (PF 411) inserted into the kidney to a depth of about 4 mm. After each experiment, the rats were killed with an overdose of the anaesthetic given i.v. and the position of the medullary probe was verified by surgically exposing the location. The laser-Doppler probes were calibrated using a motility standard (a colloidal suspension of latex particles). The Brownian motion of the suspension provides the standard value of 250 perfusion units (1,000 PU = 10 V). Thus, only relative flux values were measured but the calibration enabled comparison of the results among animals.

In order to compensate for fluid losses during the surgical preparation, a 6 % bovine albumin in isotonic saline solution was infused i.v. at 1.2 ml/h. At the end of surgical preparation and after placement of laser-Doppler probes, the infusion of 6 % albumin was replaced by one containing 2 % albumin and 7.5 % polyfructosan (Inutest, Fresenius Kabi, Linz, Austria) initiated by a priming dose of 1.6 ml/kg for 5 min and followed by a continuous infusion at 1.2 ml/h in each rat. The rats were then allowed to stabilize for 1 h.

### Experimental protocols

#### Effects of L-NAME on AP, renal haemodynamics and renal excretion and plasma ANP

After two 30-min control urine collections and measurement periods, either saline (time control group, *n* = 6) or a nonselective inhibitor of NO synthase, L-NAME (Sigma, Saint Louis, USA), was given intravenously. Urine samples were collected for six consecutive 30-min periods, and a blood sample was taken in the middle of the first control period and then repeated every 1 h. In order to evaluate the effects of the L-NAME dose on the natriuretic responses, L-NAME was infused at varying doses that ranged from 5 to 50 μg/kg/min. For further analysis, these rats were divided into two groups: one in which L-NAME did not affect AP significantly over time and the other in which AP increased after 30–45 min of infusion. In additional experiments (*n* = 4), a pressor dose of L-NAME was administered but renal perfusion pressure (RPP) was maintained at control levels using an aortic clamp placed above the left renal artery.

To check the potential effect of L-NAME on changes in circulating ANP, blood samples were collected from a separate group of rats (*n* = 5) before and after 180 min of infusion of L-NAME given i.v. at 20 μg/kg/min. The experimental protocol was similar to that described above with the exception that regional blood flows (laser-Doppler probes) in the kidney were not measured.

In another three groups of rats, anantin, a competitive antagonist of NPR-A, was infused alone (*n* = 4) at 10 μg/kg, superimposed on L-NAME infusion (*n* = 4) or co-infused with L-NAME (*n* = 6). This dose of anantin was found to be effective in the blockade of ANP-dependent diuresis in rats [6]. Anantin was found to inhibit ANP-induced intracellular cGMP accumulation by competitively binding the ANP receptor without causing guanylyl cyclase activation [26, 41]. Anantin has no agonist activity and does not inhibit NPR-C binding or activity [38]. Recently, it was found to abolish the ANP-dependent decrease in blood pressure induced by glucagon-like peptide 1 (GLP-1) receptor activation in mice [24].

After control urine collections and measurement periods and three consecutive L-NAME infusion (10 μg/kg/min) periods, 30 min each, needed to induce distinct effects of NOS inhibitors, anantin was given intravenously for 1 min; the measurements were continued for the next 180 min. The effects of anantin alone were studied using a similar protocol; however, saline vehicle was infused instead of L-NAME (*n* = 4). In the third group, instead of a bolus dose, anantin was co-infused (0.6 μg/kg/min) simultaneously with L-NAME (10 μg/kg/min) until the end of the experiment.

#### Analytical procedures

Anantin (Bachem AG, Bubendorf, Switzerland) was dissolved in 50 % acetic acid, aliquoted and stored at −20 °C. On the day of the experiment, aliquots were diluted to 10 μg in a 300-μl solution of 0.9 % NaCl [6] or added to L-NAME solution. Urine volumes were determined gravimetrically, and urine sodium and potassium concentrations were measured by flame photometry. Polyfructosan concentrations in both urine and plasma samples were measured by standard spectrophotometry. Glomerular filtration rate (GFR) was estimated from the clearance of polyfructosan.

##### ANP measurements

For the estimation of plasma ANP concentrations, blood samples were immediately centrifuged to separate the plasma. From these plasma samples, an amount of 50 μl from each sample was separated, snap-frozen in liquid nitrogen and stored at −80 °C until analysed. ANP concentration in the plasma was measured using an ANP (NPPA) rat in vitro enzyme-linked immunosorbent assay (ELISA) kit (Abcam, Cambridge, MA, USA). In this assay, 50 μl of the plasma samples as well as the ANP standards was put in each well in the plate to which was added 50 μl of biotinylated ANP antibody, and incubated for more than 2 h. The colour absorbance in these incubated samples was determined immediately on a microplate reader at a wavelength of 450 nm. The concentration of ANP in the unknown samples was determined from the standard curve generated by the regression analysis of the values from the samples of known standard concentrations. In this assay, the minimal detectable dose of ANP in the samples is ~0.2 ng/ml.

#### Statistical analysis

The significance of changes within one group over time was first evaluated by repeated measures analysis of variance (ANOVA) followed by Student’s *t* test for dependent variables. Differences in mean values between groups were analysed by one-way ANOVA followed by modified Student’s *t* test for independent variables. The standard error of the mean (SEM) was used as the measure of data dispersion. *P* < 0.05 was taken to indicate significant difference.

## Results

In the time control group, renal function remained steady and no significant changes were observed in renal haemodynamics or renal excretion parameters during the course of the experiments.

### Effects of L-NAME on AP, renal haemodynamics and renal excretion and plasma ANP

**Fig. 1:**
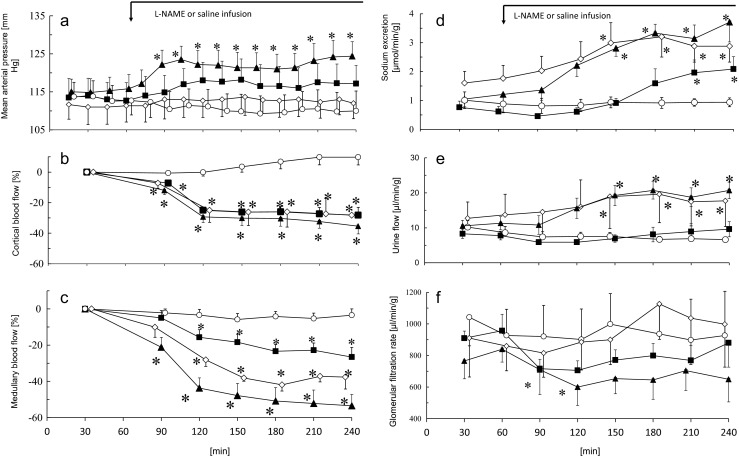
Effects of systemic L-NAME infusion on **a** mean arterial pressure (AP); **b**, **c** renal cortical and medullary blood flow (relative changes), respectively; **d** sodium excretion; **e** urine flow and **f** glomerular filtration rate. *Black squares* represent the nonpresssor lower dose of L-NAME (5–20 μg/kg/min, *n* = 5), *black triangles* the pressor higher dose of L-NAME (50 μg/kg/min of L-NAME, *n* = 5) and *white diamonds* the pressor dose of L-NAME with controlled AP (*n* = 4). *White circles* indicate the time control group (*n* = 6). Values are means ± SEM. *Asterisks* indicate significantly different vs. values before L-NAME at *P* < 0.05 or less

The lower doses of L-NAME (5–20 μg/kg/min) did not significantly increase AP (Fig. 1a) but decreased blood flow both in the cortex and medulla by 17 ± 5 % (*P* < 0.02) and 27 ± 6 % (*P* < 0.01), respectively (Fig. 1b, c). CBF and MBF were significantly reduced within 30–45 min after the start of drug administration. The slight increase in urine flow (*V*) was not significant; however, sodium excretion (U_Na_V) increased during the 3rd hour of L-NAME infusion, which was mostly due to the rise in urine sodium concentration 79 ± 11 vs. 230 ± 30 mmol/l before and after L-NAME, respectively (Fig. 1d, e). The fractional excretion of sodium increased from 0.65 ± 0.19 to 3.04 ± 1.06 % (*P* < 0.01). Urinary potassium excretion (U_K_V) was not affected by L-NAME and was 1.2 ± 0.3 vs. 1.5 ± 0.2 μmol/min before and after the drug infusion, respectively. There were no significant changes in GFR throughout the measurement periods (Fig. 1f).

With the higher dose of L-NAME (50 μg/kg/min), AP increased significantly by 30 min after the onset of the L-NAME infusion (from 116 ± 4 to 122 ± 4 mmHg, *P* < 0.001) and remained elevated throughout the experiment (124 ± 4 mmHg, *P* < 0.001). RBF changes were similar, but the decreases in CBF and MBF were greater, averaging 38 ± 5 % (*P* < 0.01) and 52 ± 6 % (*P* < 0.003), respectively (Fig. 1a–c). Parallel to the regional blood flows, total RBF also decreased by 48 ± 0.1 %, from 6.5 ± 0.9 to 3.3 ± 0.4 ml/min/g. The L-NAME-induced increase of AP was observed before the increases in urine flow and U_Na_V. These occurred during 60–90 min after the onset of L-NAME infusion and remained elevated for the duration of the experiment (Fig. 1d, e). U_K_V was similar to that observed with the lower dose and did not change before and during L-NAME infusion, 1.2 ± 0.2 vs. 1.0 ± 0.1 μmol/min/g. GFR was decreased at the beginning of the higher dose administration but was not significantly different from the control in subsequent collection periods (Fig. 1f).

**Fig. 2:**
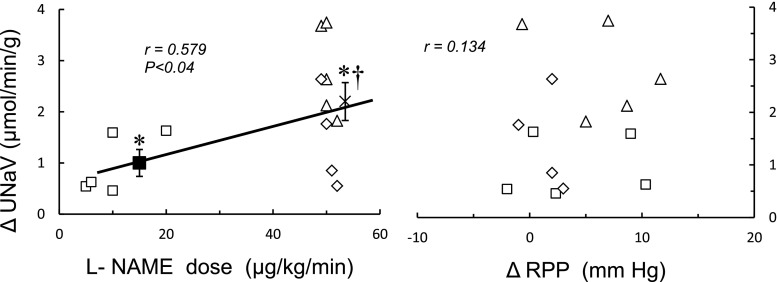
Relationships between L-NAME dose (*white squares* 5–20 μg/kg/min, *n* = 5; *white triangles* 50 μg/kg/min with uncontrolled, *n* = 5; or *white diamonds* controlled renal perfusion pressure, RPP, *n* = 4) and changes in urinary sodium excretion, U_Na_V (*left panel*) or L-NAME-induced changes in RPP (*right panel*). *X* mean value for experiments with L-NAME dose 50 μg/kg/min, *asterisks* significantly different vs. before L-NAME (*P* < 0.05 or less), *dagger* significant difference between mean value for dose 50 μg/kg/min with uncontrolled RPP and mean value for 5–20 μg/kg/min of L-NAME (*P* < 0.01). There was not a significant correlation between the change in RPP and the change in U_Na_V (*right panel*)

When RPP was controlled, the effects of the pressor dose of L-NAME on renal haemodynamics and renal excretion were similar to that observed when RPP was not controlled. The decreases in RBF (from 5.3 ± 0.6 to 3.0 ± 0.2 ml/min/g, *P* < 0.02), CBF and MBF occurred earlier and were followed distinctly later by the increases in U_Na_V and urine flow (Fig. 1). Although systemic AP above the aortic clamp increased by 18 ± 2 mmHg, AP at the level of the renal arteries was maintained within 5 mmHg of the initial pressures throughout the experiment. As in the other experimental groups, U_K_V remained stable throughout the experiment and was 0.9 ± 0.0 vs. 0.8 ± 0.1 μmol/min/g before and after L-NAME infusion, respectively. The temporal dissociation between haemodynamic and natriuretic responses in the experimental groups is shown in Fig. 1. As shown in Fig. 2, a positive correlation was found between the L-NAME dose and the relative increases in sodium excretion (*r* = 0.579, *P* < 0.04, *n* = 14). In contrast, there was not a significant relationship between the changes in RPP caused by L-NAME and the relative increases in sodium excretion (*r* = 0.134).

In the additional experimental group in which plasma ANP concentrations affected by L-NAME infusion were determined, the plasma ANP concentration increased significantly (*P* < 0.001) from 0.30 ± 0.09 before vs. 1.07 ± 0.20 ng/ml (*n* = 5) after the drug administration. This was associated with moderate AP increase, and RBF decrease occurred by 60 min after the onset of the L-NAME infusion (from 114 ± 4 to 125 ± 6 mmHg, *P* < 0.01, and 6.1 ± 1.4 to 4.6 ± 1.0 ml/min/g, *P* < 0.03, respectively). In this group, the RBF decrease (33 ± 4 %) was lower compared to that caused by a higher dose of L-NAME (−48 ± 0.1 %) but, similar to previous experiments, the increased AP and decreased RBF occurred earlier than the increases in *V* (6.6 ± 08 vs. 10.8 ± 1.9 μl/min/g, *P* < 0.03) and U_Na_V (0.5 ± 0.2 vs. 1.4 ± 0.4 μmol/min/g, *P* < 0.05) observed at the end of the experiment. Also, fractional sodium excretion increased (0.19 ± 0.09 vs. 0.74 ± 0.16 %, *P* < 0.02) despite modest decreases in GFR from 1.56 ± 0.04 to 1.25 ± 0.06 ml/min/g (*P* < 0.04) parallel AP and RBF changes. Thus, similar to the results shown in Fig. 1, the haemodynamic and natriuretic responses were temporally dissociated in this experimental group also. U_K_V did not differ before and during the drug infusion, 1.4 ± 0.2 vs. 1.2 ± 0.2 μmol/min/g, respectively.

### Effects of treatment with anantin on L-NAME-induced effects on renal excretion

**Fig. 3:**
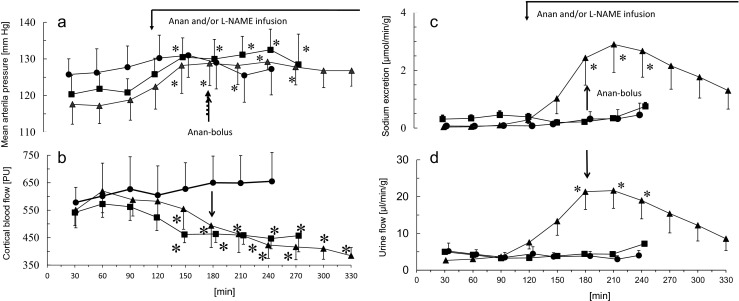
Effects of anantin (*Anan*, antagonist of natriuretic peptide receptor-A) (*black circles*) given alone (*black squares*) co-infused with L-NAME or (*black triangles*) superimposed on L-NAME (10 μg/kg/min) on changes in **a** mean arterial pressure, **b** cortical blood flow, **c** sodium and **d** urine excretion. Values are means ± SEM. *Upward arrows* represent Anan bolus (10 μg/kg) added to L-NAME. *Asterisks* indicate significantly different from values before L-NAME alone or anantin + L-NAME at *P* < 0.05 or less

Anantin, given alone, did not elicit significant changes in CBF and MBF (193 ± 34 and 160 ± 28 PU, before and after anantin, respectively) or renal excretion parameters (Fig. 3). In the group treated with L-NAME, there were increases in AP along with natriuresis and diuresis. AP was not significantly modified by anantin bolus (indicated by the arrow in Fig. 3) and was lower only by 3 ± 3 mmHg (Fig. 3a). Similarly, the decrease in RBF as measured by CBF (Fig. 3b) or RBF and MBF (6.6 ± 0.8, 3.2 ± 0.4 and 3.3 ± 0.5 ml/min/g and 134 ± 16, 105 ± 20 and 95 ± 21 PU, before and after L-NAME and after L-NAME infusion with anantin, respectively) remained unchanged. In contrast to the maintained renal haemodynamics, the urine flow and sodium excretion increases induced by L-NAME were markedly attenuated. The peak in natriuresis was gradually reduced by 57 ± 3 % (*P* < 0.004) and the peak of diuresis by 55 ± 3 % (*P* < 0.004) (Fig. 3, right-hand panel). As in the experimental groups with L-NAME alone, U_K_V was not modified throughout the experiment and was 0.8 ± 0.1, 1.3 ± 0.2 and 0.7 ± 0.2 μmol/min/g before and after L-NAME and after L-NAME infusion with anantin, respectively. Thus, the natriuretic response induced by L-NAME was markedly reduced after administration of the NPR-A blocker despite the maintained elevated AP.

Similar to the effects of bolus injections, the effects on haemodynamics of continuous infusion of both anantin and L-NAME (anantin-L-NAME co-infusion) did not modify the AP increase or CBF decrease (Fig 3a, b) and MBF decrease 121 ± 7 vs. 88 ± 10 PU before and after anantin-L-NAME co-infusion, respectively. However, during the simultaneous infusion of both drugs, there were no changes in sodium excretion or urine flow (Fig. 3c, d) that occurred when L-NAME was infused alone (Fig. 1d, e).

## Discussion

The mechanisms mediating the natriuresis in response to acute systemic infusion of NOS inhibitors have remained uncertain, but they have often been attributed to the associated increases in arterial blood pressure [10, 13, 14, 19]. In the first part of the present study, we infused the NOS inhibitor at doses that either did not affect AP significantly or produced only moderate increases. Although this experimental set-up eliminated AP as a confounding factor, it raised the question regarding the effectiveness of inhibition of NO activity. However, we clearly observed significant decreases in RBF, CBF and MBF, indicating that even lower doses of L-NAME are effective in blocking NOS activity in the kidney vasculature, even when systemic AP is not affected. The effect on MBF was more pronounced than on CBF as described previously following systemic blockade with nitro-l-arginine [4]. From the haemodynamic data, it can be concluded that the doses of L-NAME were effective in reducing intrarenal NO generation. Furthermore, the clear decrease in MBF demonstrates that the natriuresis is independent of MBF because the MBF changes would predict decreases in sodium excretion [5, 22]. Liang et al. [18] also showed natriuresis in response to a very low dose of L-NAME (1 μg/kg/min), without increases in AP or changes in GFR. They explained the natriuresis by suggesting increases in NOS in the kidney interstitium as measured by production of nitrate/nitrite. However, RBF was not measured which makes it difficult to compare with the results of the present study. Furthermore, it is unlikely that there would be a paradoxical effect of L-NAME to increase NO activity in tubular segments and hence increases in sodium excretion, even in the face of clear evidence for inhibition of NOS resulting in renal vasoconstriction and reductions in both CBF and MBF.

The present results show a positive, significant correlation between the increases in sodium excretion and the dose of L-NAME but not with the changes in AP (Fig. 2). This correlation along with the lack of associated changes in GFR indicates that the increase in sodium excretion was the result of an inhibition of tubular sodium reabsorption by mechanisms independent of pressure natriuresis. These results pose a conundrum and appear to be at variance with results from in vitro and micropuncture studies as well as clearance experiments indicating that NO is a potent inhibitor of sodium transport in renal tubules, and this effect is clearly blocked by L-NAME [19, 27]. Thus, the natriuresis observed in our experimental groups suggests that it is not simply the result of withdrawal of the NO stimulus on tubular transport but rather from an effect of NOS inhibitors on systemic factors which then affect tubular sodium reabsorption. The time difference between the changes in RBF and the changes in sodium excretion suggests that the natriuretic response has an extrarenal origin. In considering possible mechanisms, our attention was drawn to studies indicating that inhibition of NO activity augmented the release of ANP from isolated rat atrium [34]. In addition, Leskinen et al. [17] demonstrated an increase in plasma ANP in response to L-NAME infusion. On the other hand, in Wistar-Kyoto rats (WKY), L-NMMA (other nonselective NOS inhibitor) caused a marked rise in AP (30 mmHg), but plasma ANP did not change [13]. Nevertheless, contemporary cardiovascular physiology is consistent with the explanation that the systemic vascular contraction, in particular of the capacitance vessels, caused by NOS inhibitors would decrease overall vascular capacitance and increase venous return and right atrial pressure even in the absence of increases in AP, thus eliciting ANP release [33, 42]. This explanation is consistent with previous results showing that systemic NOS inhibition did not elicit natriuresis in chronic Ang II-infused rats, a condition that already has augmented vascular constriction [4]. Indeed, we have shown in the current study that systemic infusion of a NOS inhibitor at a dose which exerts moderate effects on AP or renal haemodynamics led to increases in plasma ANP concentrations which might then have increased renal sodium excretion by directly inhibiting tubular sodium reabsorption [19, 27].

The validity of our interpretation was further evaluated by administration of an ANP receptor blocker superimposed on the L-NAME-induced natriuresis and diuresis. Importantly, anantin was found to decrease the augmented urine flow and sodium excretion and return these values toward control values even in the absence of changes in either arterial pressure or CBF (Fig. 3). Additional studies demonstrated that co-infusion of anantin with L-NAME prevented the increase of sodium excretion even though AP increased and CBF decreased. When L-NAME was infused alone, the increase in sodium excretion was not abolished over the time. Collectively, these data support the conclusion that increased ANP and perhaps BNP levels in response to systemic L-NAME infusion are responsible, at least in part, for the natriuresis. It has been demonstrated that an increase in GFR is not a factor mediating ANP-induced natriuresis, at least when ANP is given at doses in the physiological range [2]. Presumably, the effects of activation of NPR-A would be additive to the effects of increased TNF-α, which has also been reported to elicit natriuresis during systemic NOS inhibition [36].

Other possible candidates that could be involved in the natriuretic response appear to be less likely. Indeed, endothelin (ET-1) in subpressor doses evokes natriuresis [11, 28, 32] despite renal vasoconstriction [11] or decreases in GFR [29] or without changes in renal haemodynamics [35]. However, it has been shown that NO is a mediator of the natriuretic effects of collecting duct-derived ET-1 [35, 37]. Accordingly, these results do not support the involvement of endothelins in mediating L-NAME-induced natriuresis.

NO inhibits renal ω-hydroxylase activity [1, 29, 39], and withdrawal of NO leads to increased ω-hydroxylase activity and expression of cytochrome P4A (CYP-4A) [30], the protein/enzyme responsible for 20-HETE synthesis. Thus, it is possible that NOS inhibition increases ω-hydroxylase activity and 20-HETE and EET synthesis and this would be expected to inhibit tubular sodium reabsorption and cause natriuresis. Increased diuresis could depend on enhanced synthesis of diuretic and natriuretic EETs and 20-HETE related to enhanced heme bioavailability for CYP-450 enzymes, the consequence of NOS inhibition. The CYP-450-dependent active agents increase sodium excretion. This influence is best observed during chronic NOS inhibition, especially in animals with high Na intake [12, 16, 40]. However, the effects of 20-HETE and EETs on renal vasculature are opposite to those observed in our experiments, because NOS inhibition reduced both CBF and MBF at all the doses used. Renal nerve activity inhibition as a mediator of natriuretic effects of systemic NO blockade can also be excluded. It was shown in WKY rats that renal denervation did not affect GFR and renal excretion responses to L-NMMA [14].

While acute changes in renal medullary blood flow have been associated with alterations in sodium and water excretion, a vasoconstrictor infused directly into the renal interstitium leads to selective reduction in MBF accompanied by a decrease of sodium and water excretion. In addition, infusion of a vasodilator leads to increases in MBF paralleled by natriuresis and diuresis. These changes occurred in the absence of alterations in CBF and AP [22]. These data support the concept that renal MBF is important in the regulation of tubular fluid and electrolyte handling; however, the mechanisms that transduce changes in medullary flow into changes in tubular sodium handling remain to be elucidated [5]. In our study, the reduction of whole kidney, cortical and MBF following administration of L-NAME was observed much earlier than the increases in sodium and water excretion and would have predicted decreases rather than increases in sodium excretion. Thus, changes of blood flow probably limited rather than contributed to the sodium excretion following NOS inhibition. Taken together, the changes in CBF and MBF probably diminished the inhibition of tubular transport and hence the magnitude of the natriuresis in response to L-NAME.

In summary, these data demonstrate that the natriuretic responses to systemic administration of L-NAME are temporally dissociated from the changes in AP and occur even under conditions of controlled arterial blood pressure and thus are not simply due to increases in arterial pressure. The observation that natriuresis related to inhibition of NOS was distinctly later in onset than were the decreases in RBF, in both cortical and medullary zones, does not support a functional association of the two phenomena. In addition, blockade of ANP receptors reversed the natriuresis when given after the L-NAME-induced natriuresis was at its peak and also prevented the natriuresis, but not the renal vasoconstriction, when it was co-infused at the same time as the L-NAME. The evaluation of the plasma ANP levels which increased significantly following administration of the systemic NOS inhibitor strengthens our conclusion. Thus, the natriuresis may be due to systemic effects of NOS inhibition, most likely by stimulating ANP, which then exerts direct tubular effects to decrease sodium reabsorption.
